# Interannual variation in nitrous oxide emissions from perennial ryegrass/white clover grassland used for dairy production

**DOI:** 10.1111/gcb.12595

**Published:** 2014-05-22

**Authors:** William Burchill, Dejun Li, Gary J Lanigan, Micheal Williams, James Humphreys

**Affiliations:** 1 Animal & Grassland Research and Innovation Centre, TeagascMoorepark, Fermoy, Co. Cork, Ireland; 2 Department of Botany, School of Natural Sciences, Trinity College DublinCollege Green, Dublin 2, Ireland; 3 Huanjiang Observation and Research Station for Karst Ecosystems, Key Laboratory of Agro-ecological Processes in Subtropical Region, Institute of Subtropical Agriculture, Chinese Academy of SciencesChangsha, Hunan, 410125, China; 4 Johnstown Castle Environment Research Centre, TeagascJohnstown Castle, Co. Wexford, Ireland

**Keywords:** freeze-thaw cycles, grazing, interannual variation, nitrous oxide, rainfall, regulating factors, white clover grassland

## Abstract

Nitrous oxide (N_2_O) emissions are subject to intra- and interannual variation due to changes in weather and management. This creates significant uncertainties when quantifying estimates of annual N_2_O emissions from grazed grasslands. Despite these uncertainties, the majority of studies are short-term in nature (<1 year) and as a consequence, there is a lack of data on interannual variation in N_2_O emissions. The objectives of this study were to (i) quantify annual N_2_O emissions and (ii) assess the causes of interannual variation in emissions from grazed perennial ryegrass/white clover grassland. Nitrous oxide emissions were measured from fertilized and grazed perennial ryegrass/white clover grassland (WC) and from perennial ryegrass plots that were not grazed and did not receive N input (GB), over 4 years from 2008 to 2012 in Ireland (52°51′N, 08°21′W). The annual N_2_O-N emissions (kg ha^−1^; mean ± SE) ranged from 4.4 ± 0.2 to 34.4 ± 5.5 from WC and from 1.7 ± 0.8 to 6.3 ± 1.2 from GB. Interannual variation in N_2_O emissions was attributed to differences in annual rainfall, monthly (December) soil temperatures and variation in N input. Such substantial interannual variation in N_2_O emissions highlights the need for long-term studies of emissions from managed pastoral systems.

## Introduction

Nitrous oxide (N_2_O) is a potent greenhouse gas (GHG) and agriculture has been identified as a major source of N_2_O accounting for 58% of global emissions, with direct emissions of N_2_O from grazed grassland systems comprising between 16% and 33% of this total (de Klein *et al.*, 2008).

Nitrous oxide is primarily produced in soil by microbial processes such as nitrification and denitrification. Other processes such as nitrifier denitrification, codenitrification and dissimilatory reduction of nitrate to ammonium (DNRA) are also known to produce N_2_O (Stevens & Laughlin, 1998). Both nitrification and denitrification are regulated by soil nitrate (NO_3_^−^), ammonium (NH_4_^+^), organic carbon (C), pH, temperature, moisture content and oxygen supply (Firestone & Davidson, 1989; Liu *et al.*, 2007; Cuhel *et al.*, 2010). Climatic conditions can directly influence these factors and, hence, N_2_O emissions (Cantarel *et al.*, 2012). For example, rainfall has a direct impact on soil moisture and oxygen status and can consequently affect N_2_O production (Luo *et al.*, 1999). Changes in these controlling factors over time create considerable temporal variation in emissions of N_2_O on a seasonal and annual basis, making it difficult to acquire representative estimates of annual N_2_O emissions.

Considerable interannual variation in N_2_O emissions has been observed in previous studies of temperate grassland (2 years) (Hyde *et al.*, 2006; Flechard *et al.*, 2007; Rafique *et al.*, 2011), native semi-arid grasslands (Du *et al.*, 2006), arable rotations (Cai *et al.*, 2013) and differing soil types (Webster & Dowdell, 1982). This variation can be driven by differences in (i) management such as stocking densities, N fertilization rates and drainage and (ii) climatic factors such as total precipitation and temperature (Du *et al.*, 2006; Flechard *et al.*, 2007; Cai *et al.*, 2013; Rees *et al.*, 2013). Despite this, the majority of N_2_O studies are focused over relatively short periods (<1 year) and are insufficient to provide robust estimates of annual N_2_O emissions or assess the variation that can occur over longer time periods.

This study measured N_2_O emissions from fertilized and grazed perennial ryegrass/white clover grassland and nongrazed perennial ryegrass grassland receiving no N input over 4 years. The objectives were to (i) quantify annual N_2_O emissions and (ii) access the causes of interannual variation in emissions from grazed perennial ryegrass/white clover grassland.

## Materials and methods

### Site description

This study was carried out at the Teagasc Solohead Research Farm (52°51′N, 08°21′W; 95 m above sea level). The predominant soils are poorly drained Gleys (90%) and Grey Brown Podzolics (10%) with a clay loam texture (29% clay, 36% silt and 34% sand) and low permeability. The soils are seasonally wet, waterlogged or flooded due to impeded drainage and a shallow water table depth (0–2.2 m below ground level) at the site. Mean soil organic carbon, total N content, bulk density and pH at 0–10 cm depth are 5.12%, 0.54%, 0.87 g cm^−3^ and 6.2, respectively. The permanent grassland at the site is used predominately for grazing and occasionally harvested for silage. The swards range from 20 to 30 years in age. This region has a temperate moist climate allowing for pasture-based dairy production with a grazing season extending to 9 or 10 months each year. Mean annual rainfall at the site (1997–2008) was 1031 mm. Mean minimum monthly soil temperature at 10 cm depth ranged from 4.3 to 8.5 °C during winter (November–January) and maximum monthly temperatures ranged from 13.9 to 18.2 °C in summer (May–July).

### Experiment design and treatments

The experiment was a randomized block design with two treatments and three replicates. The treatments were (i) grazed perennial ryegrass/white clover-based pastures (WC) and (ii) background perennial ryegrass plots (GB). The WC treatment formed part of a perennial ryegrass (*Lolium perenne* L.)/white clover (*Trifolium repens* L.)-based system of dairy production at the Solohead Research Farm which consisted of six paddocks, with an annual stocking density of 2.35 cows ha^−1^. Three of these paddocks were devoted to the WC treatment. The WC paddocks were rotationally grazed by spring calving Holstein-Friesian cows during the main grazing season (March–November) with surplus herbage occasionally removed as baled silage. Paddocks ranged in size from 1.6 to 2.1 ha with a total area of 5.4 ha and were dominated by perennial ryegrass with an annual average white clover content of herbage dry matter (DM) of 24% during the experimental period. The grazing rotation length on WC varied from 21 days in late spring/early summer to 42 days in autumn. Postgrazing sward height, maintained at 4 cm, was measured using a rising plate meter (Grasstec, Charleville, Ireland) and used to determine when cows were moved to the next paddock.

Fertilizer N and slurry applications to WC are presented in Table1. Fertilizer and slurry N were applied in a number of applications between early spring and early summer. The additional N input to WC in the form of N deposited by grazing cows was calculated based on the stocking density, residence time and N excretion per cow per day for each grazing event (Table1). Annual N excretion per cow was estimated as the difference between the cow's annual intake of N in feeds and the N output in milk and calves or in live-weight change of the cow as described in detail by Humphreys *et al*. (2008). Annual input of N for the WC paddocks due to biological N fixation was estimated to be (mean ± SE) 78.6 ± 13.2 kg N ha^−1^ in 2008/09, 112.4 ± 8.8 kg N ha^−1^ in 2009/10, 102.6 ± 10.7 kg N ha^−1^ in 2010/11 and 65.4 ± 5.6 kg N ha^−1^ in 2011/12 based on white clover contents and herbage yields using methodology described by Humphreys *et al*. (2008).

**Table 1:** Applications of fertilizer N and cattle slurry, and annual N deposited by grazing animals to perennial ryegrass/white clover pastures (WC) in 2008/09, 2009/10, 2010/11 and 2011/12

Date	Application	N input (kg ha^−1^)
2008/09		
9th February 2009	Cattle slurry	93.4
25th March 2009	Urea	28.8
8th May 2009	CAN*	34
	Excreta N†	129
2009/10		
10th February 2010	Cattle slurry	76.9
14th April 2010	Urea	57.5
	Excreta N	136
2010/11		
21st January 2011	Cattle slurry	102.9
24th February 2011	Urea	28.8
22nd March 2011	Urea	28.8
13th April 2011	Urea	28.8
	Excreta N	203
2011/12		
23rd January 2012	Cattle slurry	30.9
15th February 2012	Urea	28.8
13th March 2012	Urea	28.8
2nd May 2012	Urea	28.8
	Excreta N	171

* Calcium ammonium nitrate.

† Annual N deposited by grazing livestock.

The GB treatment was laid down to measure background soil emissions of N_2_O in the absence of N input. The area of each GB plot was 3 × 11 m and was dominated by perennial ryegrass and contained no white clover. The GB plots received no fertilizer N or cattle slurry and were not grazed. Herbage on GB was harvested by cutting during the growing season to a height of 4 cm, with clippings removed.

Both of these treatments were imposed from January 2008 and measurements began in October 2008. Therefore, each annual measurement spanned two calendar years and ran from 1st October 2008 to 30th September 2009 (2008/09), 1st October 2009 to 30th September 2010 (2009/10), 1st November 2010 to 31st October 2011 (2010/11) and 1st November 2011 to 31st October 2012 (2011/12).

### Soil and weather measurements

Daily weather data including rainfall (mm), air temperature (°C) and soil temperature at 10 cm depth (°C) were recorded at the experimental site as described by Fitzgerald & Fitzgerald (2004). Effective rainfall (rainfall minus evapo-transpiration) was determined using a water-mass balance model described by Schulte *et al*. (2005). This model is based on a modified version of the Penman–Monteith evapo-transpiration model. Input weather data included daily maximum and minimum air temperatures, rainfall, wind speed and solar radiation.

At each gas sampling, volumetric soil water content (VWC) was recorded at 0–5 cm depth using a HH2 moisture meter in conjunction with a Theta Probe sensor (Delta-T Devices, Cambridge, UK). Soil water filled pore space (WFPS) at 0–5 cm depth was calculated according to Ri *et al*. (2003).

### N_2_O flux measurements

A closed static chamber technique (Hutchinson & Moisier, 1981) was used to measure N_2_O fluxes. The chamber consisted of two parts: a permanent collar and a chamber, both of which were constructed from polyvinylchloride (PVC) pipe. The headspace height of the chamber was 40 cm with an internal diameter of 29.5 cm, resulting in a cover area of 0.0683 m^2^ and a headspace volume of 0.0273 m^3^. A 10 cm long plastic tube (internal diameter of 0.5 cm) was permanently inserted into the top of each chamber. One port of a three-way value (Discofix) was fitted to the top of this tube on the outside of the chamber. A second port of the valve was connected to a needle for extraction of gas samples from the chamber. The third port of the value could be left open when placing the chamber on the collar to prevent the build-up of air pressure within the chamber. Once the chamber was in place, this port was closed to create an air tight seal between the chamber and collar. The collars were permanently inserted into the soil to a depth of 12 cm and extended 4 cm above the soil surface following installation. The collars on WC were exposed to grazing livestock throughout the experiment. Five chambers and collars were deployed in each paddock of WC and one in each of the smaller GB plots. The N_2_O sampling was conducted (between 9:30 am and 1:00 pm) on a weekly basis with increased frequency (three times *per* week) following N applications on WC. Likewise, N_2_O sampling was conducted (between 9:30 am and 1:00 pm) on a weekly basis on GB with increased frequency (three times *per* week) following N applications on WC in 2008/09 and 2009/10. Sampling frequency on GB was lower in 2010/11 and 2011/12 and plots were sampled on five occasions in 2010/11 and on thirteen occasions in 2011/12.

On each sampling occasion, the chamber was placed on the collar. Samples were taken using a gas-tight syringe and the three-way valve fitted to the top of the chamber. Gas samples were taken immediately after chamber closure (*T*_0_) and again after 15 (*T*_15_) and 30 (*T*_30_) minutes. All gas samples were transferred to 7 ml screw-cap exetainer with Teflon-coated butyl septa (Perbio Science, Cambridge, UK). Air temperature inside the chamber and soil temperature at 5 cm depth beside the chamber was recorded using a thermo-sensor (Sensor-Tech Ltd., Castlebellingham, Ireland).

Gas samples were analysed using a gas chromatograph (GC) (Varian GC 450; Netherlands) fitted with an electron capture detector (ECD) and automatic sampler (Combi-PAL auto sampler; CTC Analytics, Zwingen, Switzerland). Column and detector temperatures were 60 and 300 °C, respectively. The N_2_O concentration (parts per million, ppm) of each sample was calculated using calibration curves. These curves were determined using N_2_O standards gases with concentrations of 0.2, 0.5, 1, 5, 10, 20, 50 and 100 ppm.

### Herbage yield and N uptake in herbage

Herbage yield was measured in each WC paddock before each grazing (or silage harvest) and concurrently on each GB plot. A 10 × 1.2 m strip was harvested to 4 cm above ground level using a rotary mower (Etesia UK Ltd, Warick, UK). The fresh herbage was weighed and a sub-sample of 100 g of the fresh herbage was dried for 24 h in an oven with forced-air circulation at 100 °C to determine DM content. A second sub-sample was stored at −20 °C before being freeze dried and milled through a 0.2 mm sieve prior to N content analysis. The N concentration in the herbage DM was determined using a Leco N analyser (LECO FP-2000; Leco Coroportiaon, St. Joseph, MI, USA). Uptake of N in herbage DM (kg N ha^−1^) was calculated using the N concentration in the herbage DM (g kg^−1^) and the DM herbage yield (kg DM ha^−1^). Nitrogen uptake in herbage DM for the GB plots was predominantly from the mineralization of soil organic matter N (MSON) and was therefore used as an indication of the difference in MSON between years but not as a direct measure of MSON. This assumes that there was little difference in the input of N to GB through dry deposition of NH_3_ between years.

### Calculations

Pre-experimental testing showed a linear increase in N_2_O concentration in the chambers over a 60 minute test period. Therefore, nitrous oxide flux calculations (g N ha^−1^ day^−1^) were calculated using the linear change in N_2_O concentrations in the chamber between *T*_0_, *T*_15_ and *T*_30_. Daily N_2_O fluxes from each WC paddock were calculated using the arithmetic means of five chambers. Cumulative N_2_O emissions were calculated by linear interpolation between sampling occasions. Global N_2_O emission factors (_G_EF) of all applied N were calculated for WC using: 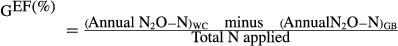


where total N applied is the sum of fertilizer N, N in cattle slurry and N deposited during grazing on WC. Total N applied was adjusted to account for losses of NH_3_ and NO_X_ assumed to be 10% from fertilizer N and 20% from N in cattle slurry and N deposited during grazing (IPCC, 2006). Biologically fixed N (BFN) was not included in the _G_EF calculations in this study as IPCC guidelines do not include BFN as a source of N_2_O (IPCC, 2006).

Annual soil N balances (SNB) for WC were calculated to determine available soil N for N_2_O emissions in each year using: 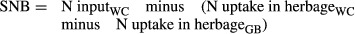


where N input is the sum of fertilizer N, slurry N, N deposited during grazing, BFN and rainfall N deposition. Fertilizer N, slurry N and N deposited during grazing were adjusted for NH_3_ volatilization as above. Rainfall N deposition was calculated by multiplying annual rainfall by rainfall total N concentration as described by (Necpálová *et al*., 2013). The N outputs from the SNB were the N removed in herbage on WC minus the background N supplied for herbage production i.e. N uptake on GB. The N removed in herbage on WC in the SNB was corrected for N uptake on GB as background supply of N is an important contributor to herbage N uptake at this site (O' Connell, 2005).

### Statistical analysis

All data were statically analysed using SAS 9.3 (SAS 2011, Institute Inc., Cary, NC, USA). Data were checked for normality by using residual normality and variance analysis. All N_2_O data required a natural log transformation [*y *= log (*x *+* *1)]. The value of one was added before log transforming the N_2_O data, which was sufficient to prevent the generation of negative transformed values. Substantially larger values of the constant added to the data may affect inference. Annual cumulative N_2_O emissions and N uptake in herbage were analysed by anova. The variables included in the model were treatment, year and the treatment by year interaction. Year was included as a repeated measure in the anova. Post hoc Holm–Tukey and Turkey–Kramer multiple comparisons test were carried out to determine differences between treatments within and between years.

Pearson correlation analysis was used to test for relationships between transformed annual N_2_O emissions and N input, weather and soil variables using data from each plot in each year (*n* = 12 for each treatment). Single and multiple linear regression (stepwise) analysis were carried out to create simple explanatory models using the same variables to account of variation in annual N_2_O emissions. A statistical probability of *P *<* *0.05 was considered significant for all statistical tests.

## Results

### Soil and weather conditions

**Fig 1:**
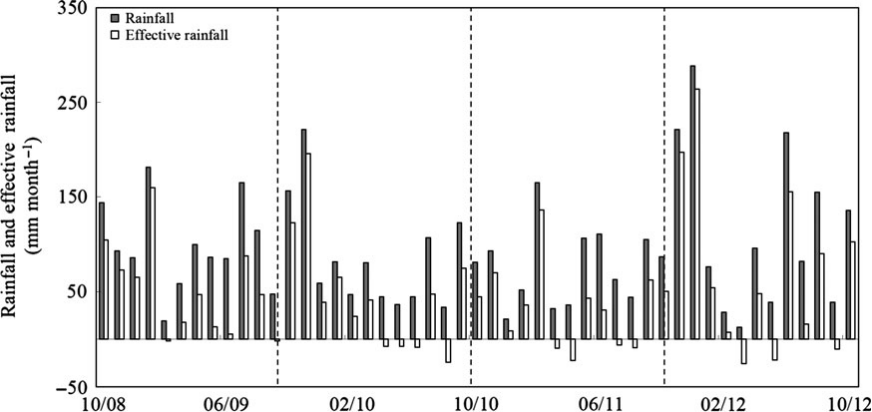
Monthly rainfall and effective rainfall (rainfall minus evapotranspiration) (mm month^−1^) at Solohead Research farm during the study period (mm/yy). Dashed vertical lines separate measurement years.

**Fig 2:**
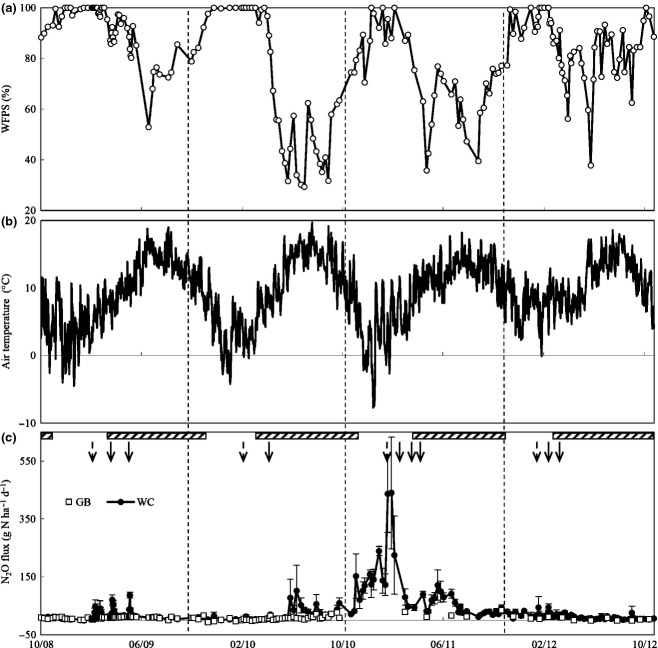
Water filled pore space (%) at 0–5 cm depth (a), daily air temperature (°C) (b) and daily N_2_O fluxes (mean ± SE) from unfertilized perennial ryegrass plots (GB) and grazed and fertilized perennial ryegrass/white clover pastures (WC) (c) during the study (mm/yy). Dashed vertical lines separate measurement years. Dashed arrows indicate dates of cattle slurry application; full arrows indicate dates of fertilizer N application. The vertical edges of the shaded boxes represent the beginning and end of each grazing season.

Annual rainfall was 1183, 1037, 919 and 1394 mm in 2008/09, 2009/10, 2010/11 and 2011/12, respectively. Monthly rainfall was higher than evapo-transpiration for much of the study particularly in 2008/09 and 2011/12 (Fig.1). Soil WFPS ranged between 30% and 100% and remained above 70% for the majority of the study. The only major exception to this was the second half of 2009/10 and the grazing season of 2010/11 (Fig.2a). Minimum and maximum mean monthly soil temperatures at 5 cm depth ranged from 3.9 to 18.3 °C, 1.9 to 18.4 °C, 1.5 to 17.1 °C and 5.4 to 17.7 °C for each of the 4 years. There was an exceptional period of cold temperatures in December of 2010/11 (Fig.2b). The mean monthly air temperature in December 2010 (0.6 °C) was much lower than typical December temperatures at this site (1950–2012 mean excluding 2010 ± SD; 6.0 ± 1.2 °C). Furthermore, the mean monthly air temperature in December 2010 was the lowest recorded between 1950 and 2012.

### Temporal trends in daily N_2_O fluxes

Nitrous oxide fluxes from WC were highly episodic with daily mean values displaying large variation throughout the study and there were clear differences in the rates of daily N_2_O fluxes both intra- and interannually (Fig.2c). Mean daily N_2_O-N emissions (g ha^−1^) from WC ranged from −0.1 to 87.3 in 2008/09, 0.8 to 101.7 in 2009/10, 12.3 to 439.8 in 2010/11 and 1.7 to 44.0 in 2011/12. Highest daily N_2_O fluxes from WC were generally found following N applications and during the main grazing season. Fluxes during winter were relatively low except for the winter of 2010/11, were peak fluxes reached 239.3 g N_2_O-N ha^−1^ d^−1^ on the 4 January 2011. Cumulative N_2_O-N emissions from WC were 9.2 kg ha^−1^ between 20 November 2010 and 20 January 2011 (period between the end of grazing season 2010 to first application of cattle slurry in 2011: Fig.2c) and equated to an 8 to 39-fold increase relative to the cumulative emissions during the same period of each of the other 3 years of the study.

The range in background emissions of N_2_O (from GB plots) was lower than WC, particularly following N applications to WC and during the grazing seasons (Fig.2c). Mean daily N_2_O-N emissions (g ha^−1^) from GB ranged from 0.5 to 14.7 in 2008/09, −2.1 to 31.5 in 2009/10, 2.0 to 35.1 in 2010/11 and −0.9 to 12.8 in 2011/12. The daily fluxes on GB were generally higher during the spring to autumn period compared with the winter.

### Annual N_2_O emissions

**Fig 3:**
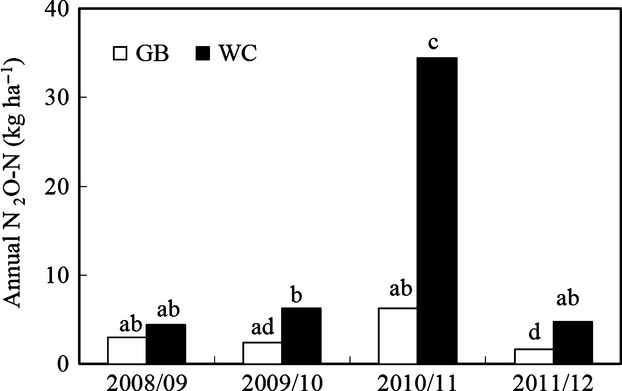
Annual N_2_O emissions from unfertilized perennial ryegrass plots (GB) and grazed and fertilized perennial ryegrass/white clover pastures (WC) in 2008/09, 2009/10, 2010/11 and 2011/12. Statistically significant differences (*P *<* *0.05) between year × treatments are indicated by different letters. Emissions on GB in 2010/11 may be underestimated as no measurements were taken during the winter of 2010, which had elevated emissions on WC.

The annual N_2_O emissions (kg N_2_O-N ha^−1^; mean ± SE) from WC ranged from 4.4 ± 0.2 in 2008/09 to 34.4 ± 5.5 in 2010/11 and from GB ranged from 1.7 ± 0.8 in 2011/12 to 6.3 ± 1.2 in 2010/11 (Fig.3). Emissions of N_2_O were affected by treatment (*P *<* *0.01), year (*P *<* *0.001) and an interaction (*P *<* *0.05) between treatment and year. There was no difference in annual N_2_O emissions between WC and GB in 2008/09, while N_2_O emissions were higher (*P *<* *0.01) from WC in each of the other years. The exceptionally high annual N_2_O emissions from WC in 2010/11 were higher (*P *<* *0.001) than emissions from WC in any of the other years, which were not different from each other.

On GB, N_2_O emissions were higher (*P *<* *0.05) in 2010/11 than in 2011/12. Otherwise, there were no differences in annual emissions from GB between years.

The annual _G_EF (mean ± SE) calculated for WC were 0.5 ± 0.2 in 2008/09, 1.6 ± 0.5 in 2009/10, 7.7 ± 1.6 in 2010/11 and 1.2 ± 0.3 in 2011/12.

### Uptake of N in herbage

**Fig 4:**
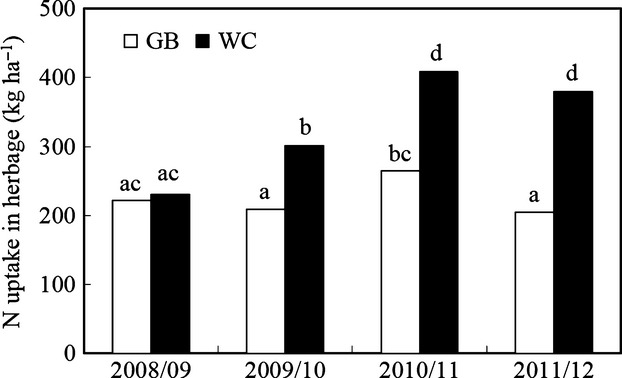
Annual N uptake in herbage dry matter on unfertilized perennial ryegrass plots (GB) and grazed and fertilized perennial ryegrass/white clover pastures (WC) in 2008/09, 2009/10, 2010/11 and 2011/12. Statistically significant differences (*P *<* *0.05) between year × treatments are indicated by different letters.

Uptake of N in herbage was affected by treatment (*P *<* *0.05), year (*P *<* *0.001) and an interaction (*P *<0.001) between treatment and year (Fig.4). Uptake of N was higher (*P *<* *0.01) on WC than GB in 2009/10, 2010/11 and 2011/12 but not different in 2008/09.

On GB, uptake of N in 2010/11 was higher (*P *<* *0.05) than in 2009/10 and 2011/12, while there was no difference in uptake between the other years (Fig.4).

### Factors affecting annual N_2_O emissions

There were significant correlations (*P *<* *0.05) between weather, soil variables and N input with annual N_2_O emissions (Table2). There was no significant correlation between annual soil N balances and N_2_O emissions from WC.

**Table 2:** Pearson correlation coefficients (*r*) between annual N_2_O emissions (*n* = 12) on unfertilized perennial ryegrass plots (GB) and grazed and fertilized perennial ryegrass/white clover pastures (WC) with annual and monthly explanatory variables

	N input§	Soil N balance††	N uptake WC	N uptake GB	Rainfall	Effective rainfall	Mean WFPS‡‡	Temp December§§	Annual Temp†††	N_2_O WC
N_2_O WC‡	0.91***	0.29NS	0.65*	0.52†	−0.74**	−0.75**	−0.71**	−0.91***	−0.65*	1
N_2_O GB‡	–	–	–	0.51†	−0.69*	−0.74**	−0.46NS	−0.74**	−0.71**	0.68*

‡ N_2_O data were transformed using the natural log transformation: ln (N_2_O+1).

§ N input = Fertilizer N + slurry N + excreta N + biologically fixed N + rainfall N deposition.

†† Soil N balance = N input minus (N uptake WC minus N uptake GB).

‡‡ WFPS = Mean annual soil water filled pore space (0–5 cm).

§§ Temp December = Mean monthly soil temperature in December (0–5 cm).

††† Annual temp = Mean annual soil temperature (0–5 cm).

† *P *<* *0.1

* *P *<* *0.05

** *P *<* *0.01

*** *P *<* *0.001, NS = not significant.

The best regression model for annual N_2_O emissions from WC included mean soil temperature in December and annual rainfall (Table3). The inclusion of annual soil temperature instead of annual rainfall also produced a robust model. Mean soil temperature in December accounted for the largest proportion of variation (highest semi-partial *R*^2^) in both of these models and hence variation in annual N_2_O emissions from WC.

**Table 3:** Multiple and single linear regressions models accounting for variation in annual N_2_O emissions from unfertilized perennial ryegrass plots (GB) and grazed and fertilized perennial ryegrass/white clover pastures (WC) using annual and monthly explanatory variables

Treatment*	Variable†	Estimate	SE	Semi-partial *R*^2^	*P* value	Model *R*^2^
WC	Temp Dec	−1.011	0.115	0.83	<0.001	0.95
	Rainfall	0.005	0.001	0.12	<0.001	
WC	Temp Dec	−0.831	0.099	0.83	<0.001	0.94
	Annual temp	1.875	0.487	0.11	<0.01	
GB	E. rain	−0.002	0.001	–	<0.01	0.55
	Temp Dec	−0.255	0.073	–	<0.01	0.55

* N_2_O data were transformed using the natural log transformation: ln (N_2_O+1).

† Temp Dec = Mean monthly soil temperature in December (0–10 cm), Rainfall = annual rainfall, Annual temp = Mean annual soil temperature (0–5 cm), N Input = Fertilizer N + slurry N + excreta N + biologically fixed N + rainfall N deposition, E. rain = annual effective rainfall, SE = standard error.

Single regression models including either effective rainfall or soil temperature in December were the best model to account for variation in annual N_2_O emissions from GB (Table3).

## Discussion

### Annual N_2_O emissions

The mean annual N_2_O-N emission of 12.5 kg ha^−1^ recorded between 2008 and 2012 for the WC pastures was very high compared to the annual average of 1.77 kg ha^−1^from multiple managed grassland sites across Europe (Flechard *et al.*, 2007). The high level of N_2_O emissions in this study were, however, similar to other Irish studies on fertilized grazed perennial ryegrass pastures receiving annual N input of between 53 and 390 kg ha^−1^ (Hyde *et al.*, 2006; Rafique *et al.*, 2011). The emissions in this study were also within the range of emissions (0.85 to 51.3 kg ha^−1^) found in fertilized grassland in the UK (Cardenas *et al.*, 2010; Rees *et al.*, 2013).

The annual background soil N_2_O-N emissions from GB were also high compared with the range of −0.5 to 1.2 kg ha^−1^ at multiple grassland sites across Europe (Flechard *et al.*, 2007) and with emissions (circa. 1 kg ha^−1^) from sandy loam-based pastures in Ireland (Abdalla *et al.*, 2009). However, emissions within the range of those reported in this study (with the exception of the high rates recorded for 2010/11) have been previously reported for gleysol-based grasslands in Ireland (−1.6 to 4.66 kg ha^−1^) (Hyde *et al.*, 2006; Rafique *et al.*, 2011).

### Global N_2_O emission factors

The _G_EF for all applied N in this study are somewhat different to IPCC EF which calculate EF individually for fertilizer N, manure N and excreta N. Similar calculations have been carried out on other N_2_O studies on grasslands, with _G_EF ranging from 0.7% to 5.1% (Hyde *et al.*, 2006; Rafique *et al.*, 2011). The reliability of the _G_EF estimates in 2010/11 and 2011/12 is questionable due to the lack of N_2_O sampling on GB, particularly over the winter of 2010/11. More intensive gas sampling on GB would have improved the accuracy of these annual _G_EF estimates and emphasizes the need for regular measurement of background emissions for accurate emission factor (EF) calculation. Nevertheless, the robust _G_EF estimates produced in 2008/09 and 2009/10 (0.5–1.6%) were relatively low compared with the other studies outlined above.

### Interannual variation in N_2_O emissions

Few published studies have reported interannual variation in N_2_O emissions from managed temperate grasslands. There was up to 7.8-fold difference in annual N_2_O emissions over the 4 years in this study. This is significantly higher than other studies on managed grassland sites in Ireland, Wales and the Netherlands where 1.1 to 3-fold differences have been reported over 2 years (Velthof *et al.*, 1996; Hyde *et al.*, 2006; Cardenas *et al.*, 2010; Rafique *et al.*, 2011). Furthermore, background emissions from the GB plots in this study also displayed up to 3.7-fold differences between years whilst previous studies reported 1.1 to 1.9-fold differences between years (Hyde *et al.*, 2006; Rafique *et al.*, 2011).

Highest emissions from both the WC and GB treatments were measured in 2010/11, which contributed to the large variation between years in this study. During 2010/11, there were exceptionally low temperatures in winter, lower annual rainfall and the highest N inputs.

### Influence of N input on N_2_O emissions

**Fig 5:**
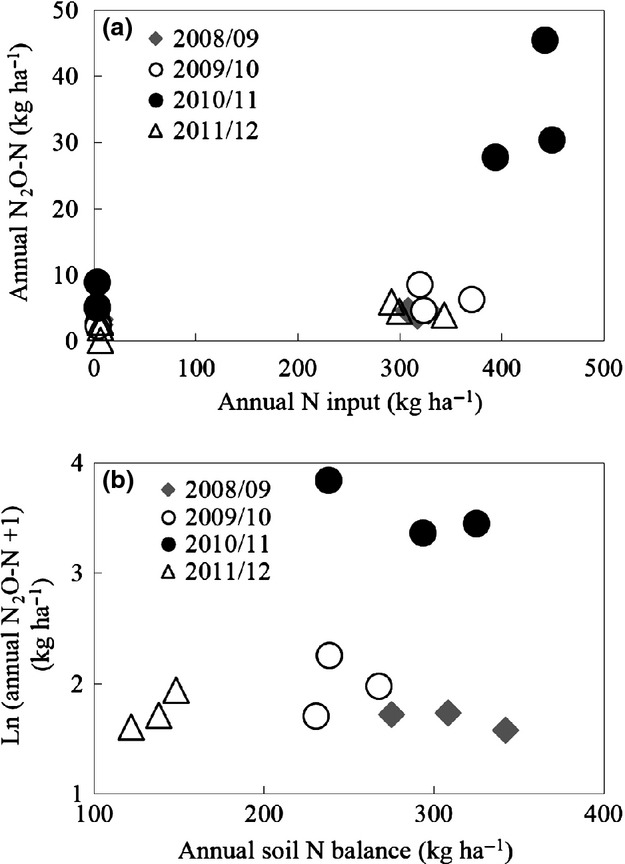
Relationship between annual N_2_O emissions and N input (a) and natural log transformed annual N_2_O emissions and soil N balances (Pearson correlation *r *=* *0.29, *P *>* *0.05) (b) using data from unfertilized perennial ryegrass plots (GB) and grazed and fertilized perennial ryegrass/white clover pastures (WC). Annual N input is the sum of fertilizer N, slurry N, N deposited during grazing, biological N fixation and rainfall N deposition. Annual N input was adjusted to account for losses of NH_3_ and NO_X_ assumed to be 10% from fertilizer N and 20% from N in cattle slurry and N deposited during grazing (IPCC, 2006).

Input of N had a large impact on annual N_2_O emissions in this study, with significantly higher emissions of N_2_O from WC compared to GB in 3 years of the study (Fig.3). Input of N also explains some of the increase in N_2_O emissions in 2010/11 relative to the other years. However, the higher N input to WC in 2010/11 (29 to 39% higher relative to other years) was relatively small compared with the large increase in annual N_2_O-N emissions (444 to 682% relative to other years; Fig5a).

Input of N, background supply of N from MSON and the rate of N removal in herbage are all crucial factors driving the availability of soil N for N_2_O emissions. Annual soil N balance for WC in 2010/11 was similar to 2008/09 and 2009/10 and indicates that despite the higher N additions in this year, the increase in N uptake in herbage on WC removed much of this additional N from the soil (Fig.5b).

All the points above indicate that alternations in N management had a significant but small contribution to the higher N_2_O emissions during 2010/11 relative to the effects of weather and soil conditions in this study.

### Influence of low soil temperatures in December

The low temperatures recorded in December 2010 were atypical of the generally mild winter conditions recorded at this site. Daily mean air temperatures were also low during periods of the winter of 2008/09 and 2009/10 (Fig.2b) but maximum daily air temperatures generally remained above zero. In contrast, maximum daily air temperature dropped below zero (minimum of −4.7 °C) for an extended period of 6 days between 20 and 25 December 2010.

Emissions of N_2_O-N during winter 2010 were up to 39 times higher than in other years of this study. The relatively cold temperatures in December 2010 are likely to have led to soil freezing and thawing, which have previously been found to stimulate large N_2_O emissions from both grassland and arable soils (Kaiser *et al.*, 1998; Kammann *et al.*, 1998; Lampe *et al.*, 2006). Increased N_2_O emissions can be attributed to the release of easily degradable N and C through the lysis of micro-organisms and disrupted soil aggregates and the reduction in N_2_O reductase activity (Christensen & Tiedje, 1990; Christensen & Christensen, 1991; van Bochove *et al.*, 2000; Holtan-Hartwig *et al.*, 2002; Müller *et al*., 2002; Mørkved *et al*., 2006). Increased emissions during thawing can be explained by the physical release of accumulated N_2_O which was produced by active micro-organisms in unfrozen soil water (Teepe *et al.*, 2001) or by a reduction in the diffusion resistance to gases that occurs during thawing (de Bruijn *et al.*, 2009).

### Influence of rainfall on N_2_O emissions

In this study, N_2_O emissions were lower under high rainfall and high WFPS (Table2). These relationships are quite different to most other studies which generally find N_2_O emissions to increase following rainfall events and with increasing WFPS (Dobbie & Smith, 2003; Liu *et al.*, 2007). Taking into account, the heavy textured soils and impeded drainage at this site, periods of high rainfall led to a shift from wet to completely saturated soil conditions in this study, whereas in many other studies it is likely that periods of high rainfall lead to a shift from dry to wet soil conditions. Saturated soil conditions (anaerobic) create more favourable conditions for complete denitrification of nitrate to dinitrogen gas, rather than nitrification and partial denitrification which would be favoured under lower WFPS (i.e. anoxic soil conditions) which were experienced in 2010/11 in this study (Monaghan & Barraclough, 1993; Rudaz *et al.*, 1999).

The relatively low rainfall in 2010/11 allowed the soils at this site to dry out more than normal, which stimulated a transition from completely saturated anaerobic to wet partially aerobic (anoxic) soil conditions (Fig.2a). Drying soil conditions is likely to have favoured net mineralization of soil organic N (Danevčič *et al*., 2010; Goldberg *et al.*, 2010), which is reflected in the increase in N uptake in herbage DM on GB in 2010/11 (Fig.3), and which provided additional substrate for nitrifying and denitrifying bacteria.

Nitrification has been found to be a major contributor to N_2_O under WFPS ranging from 40 to 60% (Stevens *et al.*, 1997; Bateman & Baggs, 2005). The reduction of WFPS to between 30% and 80% during these periods in the present study is likely to have allowed both nitrification and partial denitrification to contribute to N_2_O production simultaneously or lead to coupled nitrification–denitrification (Wrage *et al.*, 2001). This may explain some of the increase in N_2_O emissions in 2010/11 relative to other years. The results confirm that N_2_O emissions are very much increased under these anoxic soil conditions. Management decisions which support, or lead to, such soil conditions should be avoided if N_2_O emissions from grasslands, such as those in this study, are to be mitigated.

### Implications of the study

The results indicate that annual variation in N_2_O emissions from WC were regulated by weather and soil variables and N input. The dominance of weather-driven variables in regulating interannual variation has implications in the context of future climate scenarios where increased weather volatility, including temperature extremes and patterns of rainfall are predicted (Solomon *et al.*, 2007; Miraglia *et al.*, 2009).

Many studies have stressed the errors and uncertainty associated with N_2_O chamber measurements (Smith & Dobbie, 2001; Rochette & Eriksen-Hamel, 2007; Kroon *et al.*, 2008). The results of this study suggest that short-term studies may lead to bias and the underestimation or likewise the overestimation of annual N_2_O emissions. At least 2–3 years of measurement may be required to gain a representative estimate of annual N_2_O emissions from an individual site or production system which contrasts with the recommendations of Velthof *et al*. (1996). The significant contribution of the winter of 2010/11 to annual N_2_O emissions also highlights the need for frequent N_2_O measurements over winter periods even in temperate-grazed grasslands as disregarding this period could lead to underestimations of annual N_2_O emission estimates. The large variation in emissions that occurred in this study also highlights the need for regular measurement of background emissions to provide robust data for EF calculation.
